# Supplementary material for: Exploring the Contribution of Proximal Family Risk Factors on SLC6A4 DNA Methylation in Children with a History of Maltreatment: A Preliminary Study
Source: Int J Environ Res Public Health. 2021 Dec 2;18(23):12736. doi: 10.3390/ijerph182312736 (PMC8657512; doi:10.3390/ijerph182312736)
Supplement: Supplementary file 1 [file ijerph-18-12736-s001.zip › ijerph-1451894-supplementary.pdf]

Significant Correlations' Scatterplots

Table S1. Spearman correlation between the cumulative risk exposure to proximal family risk factors (CFR) and *SLC6A4* DNA methylation at CpG5.

		***SLC6A4* DNA methylation at CpG5**
CFR	*Rho*	0.467
	p	0.006
	n	33

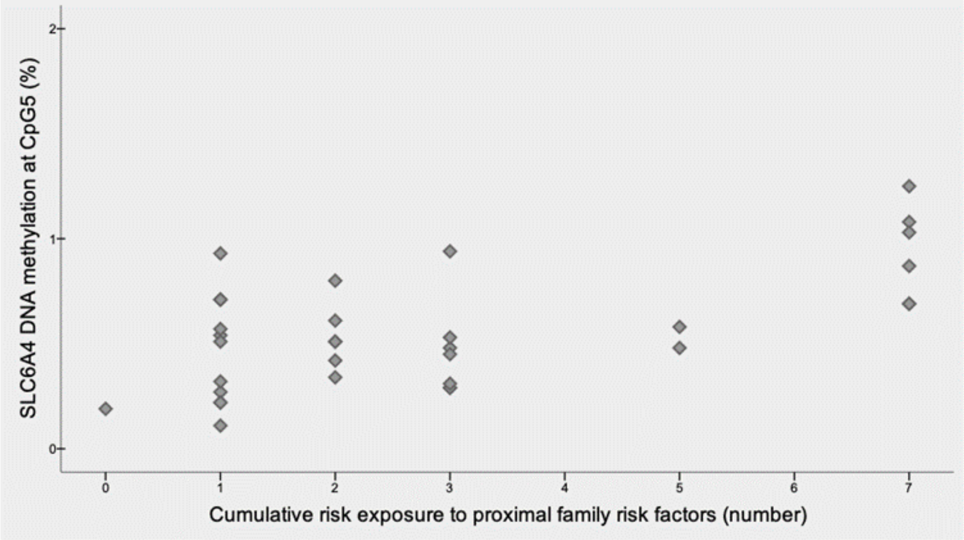

Figure S1. Scatterplots of correlation between the cumulative risk exposure to proximal family risk factors (CFR) and *SLC6A4* DNA methylation at CpG5.

Table S2. Spearman correlation between the cumulative risk exposure to proximal family risk factors (CFR) and *SLC6A4* DNA methylation at CpG13.

		***SLC6A4* DNA methylation at CpG13**
CFR	*Rho*	0.418
	p	0.016
	n	33

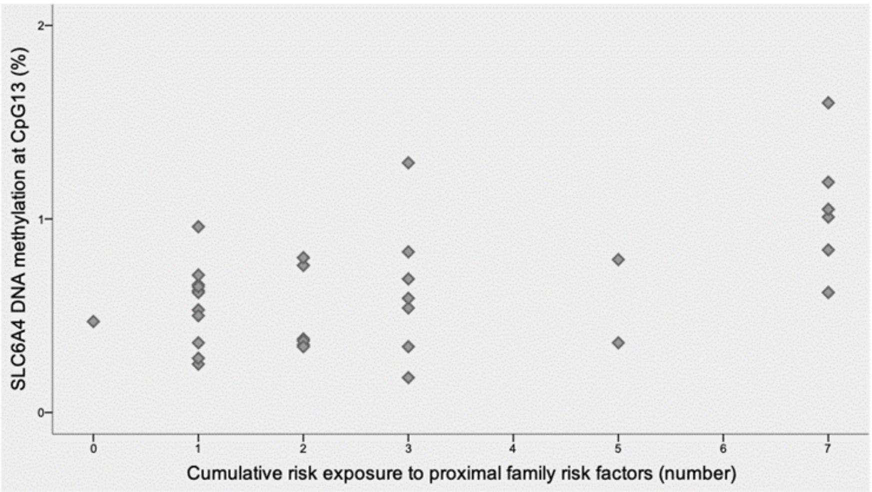

Figure S2. Scatterplots of correlation between the cumulative risk exposure to proximal family risk factors (CFR) and *SLC6A4* DNA methylation at CpG13.
